# Epidemiological Profile of the Victims of Sexual Violence Treated at a Referral Center in Southern Brazil

**DOI:** 10.1055/s-0040-1715577

**Published:** 2020-09-29

**Authors:** Michelle Dornelles Santarem, Mariane Marmontel, Nathália Lima Pereira, Letícia Becker Vieira, Ricardo Francalacci Savaris

**Affiliations:** 1 Department of Medical-Surgical Nursing, Escola de Enfermagem, Universidade Federal do Rio Grande do Sul, Porto Alegre, RS, Brasil; 2 Hospital de Clínicas de Porto Alegre, Porto Alegre, RS, Brasil; 3 Department of Gynecology and Obstetrics, Faculdade de Medicina, Universidade Federal do Rio Grande do Sul, Porto Alegre, RS, Brasil

**Keywords:** women's health service, violence against women, sexual violence, rape, serviço de saúde da mulher, violência contra a mulher, violência sexual, delitos sexuais, estupro

## Abstract

**Objective** To characterize the sociodemographic profile of women victims of sexual violence treated at a university hospital in southern Brazil.

**Method** The present cross-sectional study included all female victims of sexual violence who attended the sexual violence unit at the Hospital de Clínicas de Porto Alegre (HCPA, in the Portuguese acronym) from April 18, 2000 to December 31, 2017. Data were extracted from the electronic record of the patients and stored in a standardized questionnaire database with epidemiological aspects of the victim, the perpetrators and the type of aggression. Statistical analysis was performed using the chi-squared test for trend and descriptive statistics with 95% confidence interval (CI).

**Results** During the length of the study, 711 women victims of sexual violence were treated. The mean age of the patients was 24.4 (±10) years old (range from 11 to 69 years old) and most of the victims were white (77.4%), single (75.9%) and sought care at the unit within 72 hours after the occurrence (80.7%). In most cases, violence was exerted by a single perpetrator (87.1%), who was unknown in 67.2% of cases. Victims < 19 years old showed a higher risk of not using contraception (relative risk [RR] = 2.7; 95% CI = 1.9–3.6).

**Conclusion** Most victims of sexual violence were treated within 72 hours of the occurrence. The majority of these victims were white and young, and those < 19 years old had a higher risk of not using contraception and to know the sexual perpetrator.

## Resumo

**Objetivo** Caracterizar o perfil sociodemográfico de mulheres vítimas de violência sexual atendidas em um hospital universitário da região Sul do Brasil.

**Métodos** Estudo transversal de todas as mulheres atendidas na unidade de vítimas de violência sexual do Hospital de Clínicas de Porto Alegre (HCPA) entre 18 de abril de 2000 a 31 de dezembro de 2017. Os dados foram extraídos a partir do registro eletrônico de um questionário padronizado envolvendo aspectos epidemiológicos da vítima, do agressor e do tipo de agressão. O teste qui-quadrado foi empregado para tendência e estatística descritiva com 95% de intervalo de confiança (IC) foram utilizados para análise estatística.

**Resultados** Foram atendidas 711 mulheres vítimas de violência sexual. A média da idade das pacientes foi de 24,4 (±10) anos, sendo que a maioria das vítimas era branca (77,4%), solteira (75,9%) e buscou atendimento na unidade dentro de 72 horas após a ocorrência (80,7%). Na maioria dos casos, a violência foi exercida por agressor único (87,1%), sendo este desconhecido em 67,2% dos casos. As vítimas < 19 anos mostraram um maior risco de não estarem usando algum método contraceptivo (risco relativo [RR] = 2,7; IC95% = 1,9–3,6).

**Conclusão** A maioria das vítimas de violência sexual foi atendida dentro de 72 horas da ocorrência. As vítimas sexuais eram, na sua maioria, brancas e jovens, sendo que as < 19 anos apresentavam um maior risco de não estarem utilizando algum método contraceptivo e de conhecerem o seu agressor.

## Introduction

Violence against women is defined as “any act based on gender that causes death, harm or physical, sexual or psychological distress to women, whether in the public or the private sphere,” or as “any sexual act, attempt to obtain a sexual act, unwanted sexual comments or advances, or acts to traffic, or otherwise directed, against a person's sexuality using coercion, by any person, regardless of their relationship to the victim, in any setting, including but not limited to home and work.” 1 2 This type of violence has been a public health problem. Sexual violence can expose the victims to sexually transmitted infections, to unwanted pregnancy and to emotional problems in the short or long term, for instance, suicide and depression. 3 Twenty to 60% of the victims do not tell anyone or do not seek institutional help to report intimate partner violence. 4 The lack of official data and the underreporting problem are challenging for researchers in this area. 5 Data from specialized centers for the care of women victims of sexual abuse are scarce. 6

Homicide rates against women in Brazil have been steadily increasing since 2007, reaching 4.8 cases of female homicides/100,000 inhabitants in 2013. 7 Data from the informatics department of the Brazilian Unified Health System (DATASUS), the official electronic database of the Brazilian Ministry of Health, revealed that 243,259 domestic, sexual and/or other violence were registered in Brazil in 2016, of which 22,648 rapes were reported. 8 Around 21.9 women seek health care services for sexual violence every day and there are 14.2 reports of women victims of rape daily. 9

A study revealed that the number of police reports against women in the state of Rio Grande do Sul, Brazil, more specifically in the cities of Santa Maria, Erechim and Santana do Livramento, ranged from 66 to 361 cases between 2005 and 2009. 10 This variability in the number of police reports may be due to the systematic lack of data collection in a specialized unit for this activity. The gynecological emergency unit (GEU) of the Hospital de Clínicas de Porto Alegre (HCPA, in the Portuguese acronym) has been a reference center for the care of women victims of sexual violence since April 2000. This unit offers multidisciplinary care in emergency and outpatient settings. The staff of this unit comprises gynecologists, psychiatrists, nurses, psychologists, and social workers. The consultation for this type of victim is aimed at the prevention of sexually transmitted infections (STIs) and unwanted pregnancy. This first emergency contact is a great opportunity to offer emergency contraception for those who need it. 11 The GEU follows the Brazilian Ministry of Health protocols in this area and collects data from these victims in a systematic manner. 12 This systematic data collection may reveal some aspects of this population and the conditions in which sexual violence had occurred. The objective of the present study is to characterize the socioepidemiological profile of these victims of sexual violence who were treated at the HCPA, a university hospital in the southern region of Brazil. As a secondary objective, the average age was compared between women who were or were not using contraception at the time of violence.

## Methods

### Study Design and Setting

This is a cross-sectional study, conducted from April 1, 2000 to December 31, 2017, at the Gynecological Emergency Unit at the Hospital de Clínicas de Porto Alegre (GEU-HCPA, in the Portuguese acronym), Porto Alegre, Rio Grande do Sul, Brazil.

### Participants

Women victims of sexual assault, aged ≥10 years old, who were referred to or came spontaneously to the GEU, and had an electronic medical record were included in the study. Those without electronic records and male victims were excluded.

### Variables

Age (in years), ethnicity (by self-declaration), marital status, years of education, profession, characterization of sexual violence, that is, place where the violence occurred, number of perpetrators, characteristics of the perpetrator, whether or not there was a previous relationship between the perpetrator and the victim, form of intimidation, type of sexual assault, occurrence or not of ejaculation, first or repeated aggression, use of contraceptive method at the time of the violence, existence or not of police report and presumed age of the perpetrator were evaluated as study variables. The time elapsed between the sexual assault and the medical care and whether exams, prophylaxis, referrals for hepatitis B vaccination were provided or not were also evaluated.

### Data Sources/Measurements

After direct interviews with the patient, data were entered into an electronic medical record. Data from the electronic medical records of the patients were obtained and transferred to a specific database developed for this purpose (GoogleForms, Google LLC, Mountain View, CA, USA). Data was collected for a period of 215 months. A training period of 3 months was performed to assure the consistency of the database input. No strategy for statistical analysis was applied for missing values.

### Bias

Data were entered independently by two researchers (Marmontel M. and Santarem M. D.), which were later compared for reducing bias. Discrepancies were solved by reviewing the electronic medical record by a senior professional (Savaris R. F.) or reinterviewing the patient. Reinterview of the patient was performed either in the follow-up consultation, or by telephone by one of the authors (Marmontel M.), responsible for the outpatient clinic. In case of outdated telephone numbers, the social service of the hospital was activated.

### Sample Size

The sample was for convenience and included all cases treated within 18 years.

#### Quantitative Variables

Quantitative variables were described as means and standard deviations (SD). The population was divided into 2 groups based on a cutoff of the age most likely of not using any contraceptive method.

### Statistical Methods

Statistical analysis was descriptive using percentage and 95% confidence interval (CI), mean with SD. The identification of a cutoff of age as the most likely of not using any contraceptive method was verified by the curve receiver operator characteristics (ROC). After identifying this cutoff, the sample was divided into 2 groups for further comparisons, using the chi-squared test for trend or the Mann Whitney test. Statistical analyses were performed using the Prism 8 software (GraphPad Software, San Diego, California, USA).

### Ethical Aspects

The present study was submitted and approved by the Research Ethics Committee of the HCPA (CAAE = 84939318000005327).

## Results

### Participants

Between April 18, 2000 and December 31, 2017, a total of 711 female victims of sexual violence (100%) were screened for consultation and entered in the analysis. There were no exclusions.

### Descriptive Data

The mean (SD) and median age of the studied population were 24.1 (±10) years old and 22 years old, respectively, ranging from 11 to 69 years old. Further details of the population characteristics, the characteristics of violence and the provided care given at the first visit are described in Tables 1 , 2 and 3 , respectively.

**Table 1:** Sociodemographic characteristics of women victims of sexual violence

Variable	Overall ( *n* = 711)		≤ 19 years old ( *n* = 262)		> 19 years old ( *n* = 449)		*p-value* *
	n (%)	95%CI	n (%)	95%CI	n (%)	95%CI	
Ethnic group							
White	550 (77.4)	74.1–80.3	194 (74)	68.4–79.0	356 (79.3)	75.3–82.8	0.08
Non-white	154 (21.6)	18.8–24.8	64 (24.4)	19.6–3.0	90 (20)	16.6–24.0	
Ignored	7 (1.0)	0.5–2.1	4 (1.5)	0.6–4.0	3 (2.1)	0.2–2.1	
Marital Status							
Single	540 (75.9)	72.7–79.0	248 (94.7)	91.2–96.8	292 (65.0)	60.5–69.3	< 0.001
Married	100 (14.1)	11.7–16.8	7 (2.7)	1.3–5.5	93 (20.7)	17.2–24.7	
Separated or Widow	64 (9.0)	7.1–11.3	1 (0.4)	0.1–2.7	63 (14)	11.1–17.6	
Ignored	7 (1.0)	0.5–2.1	6 (2.3)	1.0–5.0	1 (0.2)	0–1.6	
Education							
Illiterate	9 (1.3)	0.7–2.4	2 (0.8)	0.2–3.0	7 (1.6)	0.7–3.2	< 0.001
≤ 9 years	256 (36.0)	32.5–39.6	137 (52.3)	46.2–58.3	119 (26.5)	22.6–30.8	
10–12 years	299 (42.1)	38.5–45.7	96 (36.6)	31.0–36.6	203 (45.2)	40.7–49.9	
≥ 13 years	87 (12.2)	10.0–14.9	2 (0.8)	0.2–3.0	85 (18.9)	22.8–15.6	
Ignored	60 (8.4)	6.6–10.7	25 (9.5)	6.5–13.8	35 (7.8)	5.6–10.7	
Employment status							
Unemployed	71 (10)	8.0–12.4	12 (4.6)	2.6–7.8	59 (13.1)	10.3–16.6	< 0.001
Employed	223 (31.4)	28.1–34.9	22 (8.4)	5.6–12.4	201(44.8)	40.2–49.4	
Student	218 (30.7)	27.4–34.2	154 (58.8)	52.7–64.6	64 (14.3)	11.3–17.8	
Ignored	199 (28.0)	24.8–31.4	74 (28.2)	23.1–34.0	125 (27.8)	23.9–32.2	

Abbreviation: CI, confidence interval.

* Chi-squared for trend comparing groups ≤ 19 and > 19 years old only.

**Table 2:** Characteristics of the sexual violence in the studied population and by the age of 19 years old

Variable	Overall ( *n* = 711)		≤ 19 years old ( *n* = 262)		> 19 years old ( *n* = 449)		*p-value* *
	n (%)	95%CI	n (%)	95%CI	n (%)	95%CI	
First occurrence							
Yes	525 (73.8)	70.5–76.9	201 (76.7)	71.2–81.5	324 (72.2)	67.8–76.1	
No	37 (5.2)	3.8–7.1	16 (6.1)	3.8–9.7	21 (4.7)	3.1–7.1	0.1
Ignored	149 (21.0)	18.1–24.1	45 (17.2)	13.1–22.2	104 (23.2)	19.5–27.3	
Relationship with perpetrators							
Unknown	478 (67.2)	63.7–70.6	152 (58.0)	51.9–63.9	326 (72.6)	68.3–76.5	
Known	207 (29.1)	25.9–32.6	99 (37.8)	32.1–43.8	108 (24.1)	20.3–28.2	<0.001
Ignored	26 (3.7)	2.5–5.3	11 (4.2)	2.3–7.4	15 (3.3)	2.0–5.5	
Place of occurrence							
Street	87 (12.2)	10.0–14.9	27 (10.3)	7.2–14.6	60 (13.4)	10.5–16.8	
Residence	166 (23.3)	20.4–26.6	67 (25.6)	20.6–31.2	99 (22.0)	18.4–26.1	
Work	19 (2.7)	1.7–4.2	2 (0.8)	0.2–3.0	17 (3.8)	2.4–6.0	0.4
Other	44 (6.2)	4.6–8.2	13 (5.0)	2.9–8.4	31 (6.9)	4.9–9.7	
Ignored	395 (55.6)	51.9–59.2	153 (58.4)	52.3–64.2	242 (53.9)	49.3–58.5	
Number of perpetrators							
Single	619 (87.1)	84.4–89.3	232 (88.5)	84.1–91.9	387 (86.2)	82.7–89.1	
Multiple	73 (10.3)	8.2–12.7	20 (7.6)	5.0–11.5	53 (11.8)	9.1–15.1	0.8
Ignored	19 (2.7)	1.7–4.2	10 (3.8)	2.7–7.0	9 (2.0)	1.0–3.8	
Form of Aggression							
Physical	139 (19.5)	16.8–22.6	44 (16.8)	12.7–21.8	95 (21.2)	17.6–25.2	
Verbal	48 (6.8)	5.1–8.9	15 (5.7)	3.5–9.3	33 (7.3)	5.3–10.2	0.3
More than one type	16 (2.3)	1.4–3.6	4 (1.5)	0.6–4.0	12 (2.7)	1.5–4.7	
Ignored	508 (71.4)	68.0–74.7	199 (76.0)	7.4–80.8	309 (68.8)	64.4–72.9	
Type sexual contact							
Oral	15 (2.1)	1.3–3.5	6 (2.3)	1.0–5.0	9 (2.0)	1.0–3.8	
Anal	22 (3.1)	2.0–4.7	9 (3.4)	1.8–6.5	13 (2.9)	1.7–4.9	
Vaginal	366 (51.5)	47.8–55.1	155 (59.2)	53.1–65.0	211 (47.0)	42.4–51.6	0.01
More than one type	244 (34.3)	30.9–37.9	70 (26.7)	21.7–32.4	174 (38.8)	34.3–43.4	
Ignored	64 (9.0)	7.1–11.3	22 (8.4)	5.6–12.4	42 (9.4)	7.0–12.4	
Ejaculation							
Yes	344 (48.4)	44.7–52.1	121 (46.2)	40.2–52.3	223 (49.7)	45.0–54.3	
No	25 (3.5)	2.4–5.2	7 (2.7)	1.3–5.5	18 (4.0)	2.5–6.3	0.2
Ignored	342 (48.1)	44.4–51.8	134 (51.1)	45.1–57.2	208 (46.3)	41.7–51.0	
Approximate age of the perpetrators							
≤ 20 years old	21 (3.0)	1.9–4.5	5 (1.9)	0.8–4.5	16 (3.6)	2.2–5.7	
21–30 years old	79 (11.1)	9.0–13.6	28 (10.7)	7.5–15.1	51 (11.4)	8.7–14.6	
31–40 years old	40 (5.6)	4.2–7.6	11 (4.2)	2.3–7.4	29 (6.5)	4.5–9.1	
41–60 years old	18 (2.5)	1.6–4.0	9 (3.4)	1.8–6.5	9 (2.0)	1.0–3.8	0.2
Ignored	553 (77.8)	74.6–80.7	209 (79.8)	74.5–84.2	344 (76.6)	72.5–80.3	
Police Report							
No	39 (5.5)	4.0–7.4	13 (5.0)	2.9–8.4	26 (5.8)	4.0–8.4	
Yes	515 (72.4)	69.0–75.6	203 (77.5)	72.0–82.1	312 (69.5)	65.1–73.6	0.1
Ignored	157 (22.1)	19.2–25.3	46 (17.6)	13.4–22.7	111 (24.7)	20.9–28.9	

Abbreviation: CI, confidence interval.

* Chi-squared for trend comparing groups ≤19 and > 19 years old only.

**Table 3:** Description of emergency care given to the women after sexual violence

Variable	Overall ( *n* = 711)		≤ 19 years old ( *n* = 262)		> 19 years old ( *n* = 449)		*p-value* *
	n (%)	95%CI	n (%)	95%CI	n (%)	95%CI	
Presence of physical injuries							
Yes	13 (1.8)	1.1–3.1	5 (1.9)	0.8–4.5	8 (1.8)	0.9–3.5	
No	527 (74.1)	70.8–77.2	187 (71.4)	65.6–76.5	340 (75.7)	71.5–79.5	0.2
Ignored	171 (24.1)	21.0–27.3	70 (26.7)	21.7–32.4	101 (22.5)	18.9–26.6	
Prophylaxis for sexually transmitted diseases dispensed							
Yes	612 (86.1)	83.3–88.4	232 (88.5)	84.1–91.9	380 (84.6)	87.7–81.0	
No	10 (1.4)	0.8–2.6	4 (1.5)	0.6–4.0	6 (1.3)	0.6–2.9	0.1
Ignored	89 (12.5)	10.3–15.2	26 (9.9)	6.8–14.2	63 (14.0)	11.1–17.6	
Prophylaxis for HIV dispensed							
Yes	621 (87.3)	84.7–89.6	231 (88.2)	83.7–91.6	390 (86.9)	83.4–89.7	
No	40 (5.4)	4.2–7.6	15 (5.7)	3.5–9.3	25 (5.6)	3.8–8.1	0.5
Ignored	50 (7.0)	5.4–9.2	16 (6.1)	3.8–9.7	34 (7.6)	5.5–10.4	
Referral for Hepatitis B Vaccine							
Yes	609 (85.7)	82.9–88	227 (86.6)	81.9–90.3	382 (85.1)	81.5–88.1	
No	52 (7.3)	5.6–9.5	19 (7.3)	4.7–11.1	33 (7.3)	5.3–10.2	0.4
Ignored	50 (7.0)	5.4–9.2	16 (6.1)	3.8–9.7	34 (7.6)	5.5–10.4	
Immunoglobulin Hepatitis B dispensed							
Yes	116 (16.3)	13.8–19.2	49 (18.7)	14.4–23.9	67 (14.9)	11.9–18.5	
No	545 (76.7)	73.4–79.6	197 (75.2)	69.6–80.1	348 (77.5)	73.4–81.1	0.1
Ignored	50 (7.0)	5.4–9.2	16 (6.1)	3.8–9.7	34 (7.6)	5.5–10.4	
Emergency contraception dispensed							
Yes	389 (54.7)	51.0–58.3	181 (69.1)	63.2–74.4	208 (46.3)	41.7–51.0	
No	272 (38.3)	34.7–41.9	65 (24.8)	19.9–30.4	207 (46.1)	41.5–50.7	<0.001
Ignored	50 (7.0)	5.4–9.2	16 (6.1)	3.8–9.7	34 (7.6)	5.5–10.4	
Victim was pregnant							
Yes	13 (1.8)	1.1–3.1	2 (0.8)	0.2–3.0	11 (2.4)	1.4–4.4	
No	454 (63.9)	60.2–67.3	162 (61.8)	55.8–67.5	292 (65.0)	60.5–69.3	0.09
Ignored	244 (34.3)	30.9–37.9	98 (37.4)	31.7–43.4	146 (32.5)	28.3–37.0	
Sought consultation within 72h of the violence							
Yes	574 (80.7)	77.7–83.5	214 (81.7)	76.5–85.9	360 (80.2)	76.2–83.6	
No	117 (16.5)	13.9–19.4	41 (15.6)	11.7–20.6	76 (16.9)	13.7–20.7	0.6
Ignored	20 (2.8)	1.8–4.3	7 (2.7)	1.3–5.5	13 (2.9)	1.7–4.9	
Referral to psychiatrist							
Yes	259 (36.4)	33.0–40.0	93 (35.5)	29.9–41.5	166 (37.0)	32.6–41.5	
No	272 (38.3)	34.7–41.9	96 (36.6)	31.0–42.7	176 (39.2)	34.8–43.8	0.3
Ignored	180 (25.3)	22.2–28.7	73 (27.9)	22.8–33.6	107 (23.8)	20.1–28.0	
Follow-up at the gynecology outpatient clinic							
Yes	497 (69.9)	66.4–73.2	181 (69.1)	63.2–74.4	316 (70.4)	66.0–74.4	
No	208 (29.3)	26.0–32.7	78 (29.8)	24.5–35.6	130 (29.0)	24.9–33.3	0.6
Ignored	6 (0.8)	0.4–1.9	3 (1.1)	0.4–3.5	3 (0.7)	0.2–2.1	

Abbreviation: CI, confidence interval.

* Chi-squared for trend comparing groups ≤19 and > 19 years old only.

### Outcome Data

**Fig. 1:**
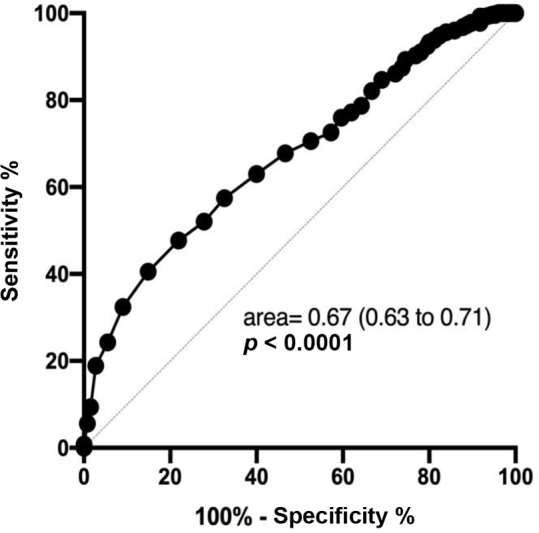
ROC curve plotting age and use or not of any contraceptive method during the occurrence of sexual violence.

The median age of the victims using contraception ( *n* = 215) was significantly higher compared with those not using any method ( *n* = 496) (24 versus 20; *p* < 0.0001; Mann Whitney test). In the ROC curve analysis ( Fig. 1 ), the cutoff point for identifying women with a higher risk of not using contraception was 19 years old. From a total of 262 women ≤ 19 years old, only 61 were using some contraceptive method, (23.3%; 95%CI: 18.6–28.8%). In contrast, 449 women were >19 years old; from these, 173 were using some contraceptive method (38.5%; 95%CI: 34.0–43.2%). These figures give a relative risk (RR) = 2.7 (95%CI = 1.96–3.6).

### Main Results/Other Analyzes

The subgroup analysis of the population revealed that most of the perpetrators were known by the victims ≤ 19 years old and the vaginal contact was more common in this subgroup, while those > 19 years old suffered more than one sexual contact and the majority of the women > 19 years old were married or with a previous relationship ( Table 1 ). Women ≤ 19 years old received more emergency contraception (69.1%; 95%CI= 63.2–74.4%). Most victims of violence sought care within 72 hours of occurrence (574 out of 711 cases, 80.7%; 95%CI = 77.7–83.5%) and continued follow-up (497 out of 711 [69.9%]; 95%CI = 66.4–73.2%) at the gynecology outpatient clinic.

## Discussion

The victims of sexual violence treated at the HCPA between 2000 and 2017 had a mean age of 24.4 years old. Those between 18 and 25 years old comprised 25.7% of the studied population (183 out of 711; 25.7%; 95%CI= 22.7–29.1%). Our data are different from the British data. Data from a referral center for sexual abuse cases in the UK showed that 50% (95%CI = 46.9–53.6%) of the cases were aged between 18 and 25 years old. 13 Our results also differ from those reported in DATASUS for 2017, either for Brazil (36.5%) or for the state of Rio Grande do Sul (32.5%). This discrepancy could be explained by the age distribution in the city of Porto Alegre, where 13% of the female population is between 15 and 29 years old. 14

The main ethnic group in our cohort was white (77.4%; 95%CI = 74.1–80.3%). This finding follows the DATASUS (2017) data; the majority of victims of violence in the state of Rio Grande do Sul are white (78.4%; 16,962 out of 21,639). 15 This is explained by the epidemiological profile of women from our state; from a universe of 5.4 million women, 83.2% are white. 16

From our data, it was possible to verify that only 31.1% (221 of 711; 95%CI = 27.7%–34.6%) of the victims of violence were using some contraceptive method. This information is relevant and there are scant data to be compared. Most of the studies use the numbers of unwanted pregnancies as proxy. 17 18 19

Most victims of violence were assaulted by unknown perpetrators (67.2%; 95%CI = 63.7–70.6%) in their residence (23.3%; 95%CI = 20.4%–26.6% [ Table 2 ]), which is in line with data presented by Delziovo et al 20 in a sample of a public service in southern Brazil, in the state of Santa Catarina. Most victims of violence sought care within 72 hours after the occurrence (80.7%; 95%CI = 77.7–83.5% ( Table 3 ). There was a low incidence of physical injuries in these victims ( Table 3 ). These findings are different from other authors, 21 but they are in agreement with those found in a Danish cohort, where they reported a 2% incidence of physical injury. 22 The low incidence of physical injuries does not allow us to find a plausible explanation. Some authors explain it by the degree of resistance by the victim, 23 while others explain this finding by the paralysis presented by the victim during the sexual assault. 24 Both explanations seem valid but we are not able to perform such analysis.

Analysis of the ROC curve showed that abused women < 19 years old had a 2.7 higher risk of not using any contraceptive method, compared with older women. This information is new and reveals the importance, for health professionals, to evaluate the contraceptive method used by the victim. Patel et al 25 published that only 40% of emergency departments offer counseling and provision of emergency contraceptives.

After dividing the sample by the age of 19 years old, some significant differences were found, such as the type of sexual contact with the victims ( Tables 1 and 3 ). The majority of women > 19 years old had a previous or current relationship, had > 10 years of education and were employed. In contrast, most women ≤ 19 years old were single, had < 9 years of education and were students ( Table 1 ). These findings are expected, since these social events, for example, to be married, are more frequent in older women. According to the literature, the younger the women who suffered sexual assault, the higher the incidence of psychologic and physical abnormalities in the future. 26

The prevalence of known perpetrators was higher among women ≤ 19 years old, compared with those > 19 years old ( Table 2 ). This finding is in accordance with the data provided by Rapee, Abuse & Incest (RAINN), an American anti-sexual violence organization (rainn.org). According to RAINN, 80% of the rapes are committed by someone known to the victim. 27 Similar results were presented by Sodipo et al 28 in Nigeria. Other authors have reported that rapists can be friends, colleagues or family members 29 30 but they do not mention a difference in age. Possible explanations for this association can be related to cultural aspects of the community and the abusive behavior of the perpetrator, associated with the economic dependency of the victim. 29 Further research is necessary on this topic.

The present study has some limitations. The sample is limited to one region in southern Brazil. A significant proportion of information, such as the location of the occurrence, the form of aggression, if ejaculation occurred, the age of the rapist and the form of violence ( Tables 2 and 3 ) were lacking, jeopardizing further analysis. Some variables, for instance, the age and number of rapists, were impossible to obtain from the history of the patient; many women were drugged or intoxicated and they were not able to recall the events. Others were traumatized and did not want to tell the details. However, although these variables were missing, others, from the same patient, were present, such as relationship with perpetrators and marital status, which had 99% of completeness. Thus, caution is required for interpreting our results on these variables.

A positive aspect of the present study is its 17-year span. This cohort presents data from the region of Porto Alegre, the city with the highest number of notifications in Rio Grande do Sul, according to the DATASUS. 15 Efforts were made to minimize the inherent biases of this type of study, such as double-checking the data and active search with the patient in outpatient follow-up.

## Conclusion

The victims of violence seen at the HCPA were mostly white, with a mean age of 24.4 years old. Those < 19 years old had a higher RR: 2.7 (95%CI = 1.96–3.6) of not using contraception, and the majority of the perpetrators are known by these young women. Health professionals must provide emergency contraception to these victims, mainly to those < 19 years old. Emergency contraception is more effective before 72 hours and most victims seek care within 72 hours of the occurrence. Finally, the relationship with the perpetrator should be investigated and proper measures must be taken when the victim knows the perpetrator.
